# Chronic nonpuerperal uterine inversion and necrosis: a case report

**DOI:** 10.1186/1752-1947-4-381

**Published:** 2010-11-25

**Authors:** Charlie C Kilpatrick, Lubna Chohan, Robert C Maier

**Affiliations:** 1 UTHealth, Department of Obstetrics and Gynecology, Lyndon Baines Johnson Hospital, 5656 Kelley Street, Houston, TX 77026, USA; 2 UTHealth, Department of Obstetrics and Gynecology, Memorial Hermann Hospital, 6431 Fannin Street Suite 3.272, Houston, TX 77030, USA

## Abstract

****Introduction**:**

Inversion of the non-pregnant uterus is rare.

****Case presentation**:**

A 56-year-old African American woman presented to our emergency center with complaints of a mass protruding from her vagina. She subsequently underwent vaginal myomectomy, abdominal hysterectomy and bilateral salpingo-oophorectomy. Pathologic examination revealed a necrotic fibroid and endometrium. At the time of laparotomy an inverted uterus was diagnosed when a 3 cm dimple containing bilateral round ligaments, infundibulopelvic ligaments and bladder was observed.

**Conclusion:**

Chronic nonpuerperal inversion of the uterus is rare. Infection should be suspected and appropriate broad spectrum antibiotics begun while planning surgery. An attempt at vaginal restoration and removal is difficult. Abdominal hysterectomy may be necessary taking care to locate the distal urinary collecting system.

## Introduction

Chronic uterine inversion of the nonpuerperal uterus is an uncommon event, reported approximately 100 times in the literature since 1940 [1,2]. Chronic nonpuerperal uterine inversion is often associated with uterine pathology. Prolapsed fibroids tend to be the most common inciting factor with occasional reports of inversion associated with uterine neoplasm and endometrial polyps [1,3-6]. Three contributing factors proposed for uterine inversion are 1) sudden emptying of the uterus which was previously distended by a tumor 2) thinning of the uterine walls due to an intrauterine tumor, and 3) dilatation of the cervix [3]. The following is a case report of a woman who presented hospital with nonpuerperal uterine inversion secondary to a prolapsed necrosing submucous fibroid, with accompanying uterine necrosis.

## Case presentation

A 56-year-old African American woman presented to our emergency center reporting that for approximately one year she had noticed a 'tangerine'-sized mass that extruded from her vagina approximately one to two times per week. This occurred sporadically and initially the mass was easily reducible. Prior to the mass coming out she would experience lower back pain, and noticed that sometimes prolonged periods of standing or walking would precipitate the event. She described the pain as 8 out of 10 when the mass was extruded, crampy in nature and sometimes associated with nausea.

When the mass could no longer be reduced she sought attention from a physician. The mass was sometimes associated with slight bleeding, and recently had developed a foul odor. She denied any difficulty voiding, weight loss, change in appetite, fevers, or chills. She did report constipation.

She had three term vaginal deliveries, a medical history of chronic hypertension of five years, and sarcoidosis mainly affecting her lungs for which she used an inhaled steroid, fluticasone. She was menopausal for four years and denied any history of sexually transmitted diseases, abnormal pap smears and other postmenopausal bleeding. She had no significant past social history and no known drug allergies.

She was afebrile, and her other vital signs were within normal limits. She had slightly atrophic external female genitalia with normal appearing labia; the urethral meatus could not be seen.

There was an approximately 10 cm well circumscribed mass, thought likely to be a fibroid, protruding four centimeters (cms) past the hymenal ring. It was beefy red in appearance; there was no active bleeding, and there appeared to be a thick base to the mass. The cervix could be palpated from approximately two to five o'clock, but could not be seen. The discernible uterus was not palpable on rectovaginal exam. An attempt was made at straight catheterization of the bladder but the urethral meatus could not be located. Ultrasound revealed an empty bladder post voiding.

**Figure 1:**
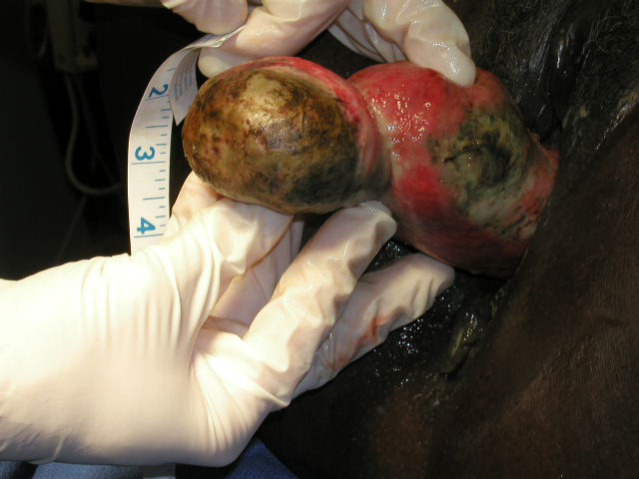
**Prolapsed uterine fibroid attached at the uterine fundus**. Notice the areas of necrosis on the fibroid as well as the endometrial lining, inverted in this case. The tubal ostia could not be visualized.

**Figure 2:**
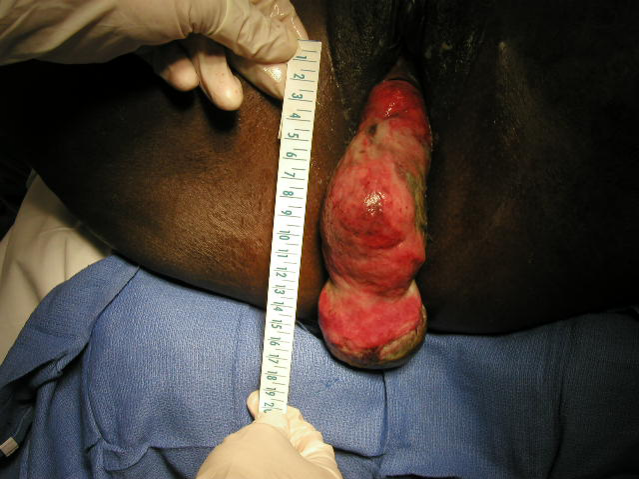
**Vaginal views of the fibroid and inverted uterus, highlighting the size of the mass, and its necrotic nature**.

The differential diagnosis included a prolapsed uterine fibroid, uterine sarcoma, endometrial cancer, and endometrial polyp. The patient was discharged with an appointment in the clinic. Upon presentation an approximately 8 cm fibroid was prolapsed out of the vagina, 15 cms past the introitus attached to what appeared to be an inverted uterus. No discernible cervix could be seen or felt on exam, and the patient did not tolerate the examination well. An area of necrosis approximately 4 cm in diameter was observed and the mass was foul smelling (Figure 1). We could not identify the ostia of the fallopian tubes. We suspected inversion of the uterus secondary to the large prolapsed fibroid, and were concerned about the area of necrosis on the fibroid and uterus. We admitted our patient to the hospital, started broad spectrum antibiotics, ordered imaging of the pelvis and biopsied the mass. Three days later her biopsies returned without signs of cancer. We imaged the pelvis preoperatively revealing the right but not the left lower collecting system. Preoperative hemoglobin and hematocrit were 9.7 and 30.0 respectively. Vaginal views of the mass are shown in Figure 2.

**Figure 3:**
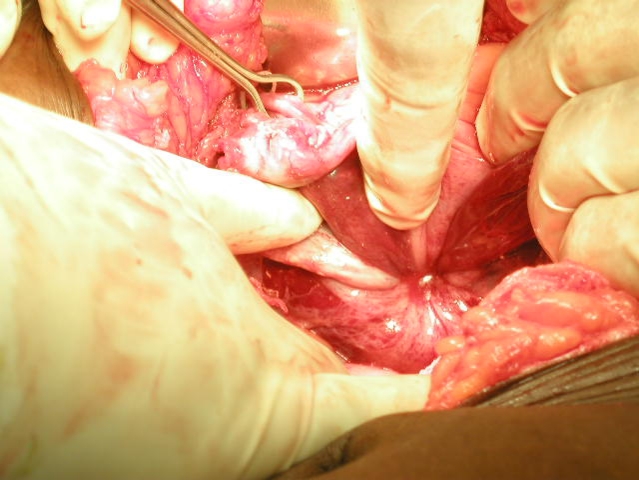
**Inverted uterus visualized abdominally**. Notice the Babcock Clamp on one of the ovaries. The finger at the 12 o'clock position is pointing at one of the round ligaments.

We performed a vaginal myomectomy with the goal of restoring the inverted uterus to its normal anatomic state prior to proceeding with a vaginal hysterectomy. Under general anesthesia with a halogenated anesthetic agent we unsuccessfully attempted to replace the uterus. Therefore we decided to open the abdomen. After packing the bowel away we inspected the pelvis (Figure 3). This is an abdominal view of an inverted uterus noting bilateral round ligaments and utero-ovarian ligaments drawn into an approximate 3 cm circle. We attempted to follow the round ligaments into the circle as classically described by Huntington with pressure exerted from the vagina but were not successful [7]. The constricting ring was too tight. After identifying both ureters, we performed Haultain's procedure followed by abdominal hysterectomy and bilateral salpingo-oophorectomy [8]. The patient was discharged on the third postoperative day and no problems were identified on two post-discharge clinic visits. The pathology report noted a 40 gram uterine leiomyoma with extensive necrosis and a 170 gram uterus with microscopic evidence of endometrial necrosis and inflammation extending into the myometrium.

## Discussion

There are over a hundred case reports of chronic nonpuerperal inversion of the uterus. Most reports cite a prolapsed fibroid as the cause, with some reporting that the fibroid was infected [3]. We are unaware of a report which documents necrosis of the prolapsed fibroid and endometrium with inflammation in the surrounding myometrium.

The uterus when fully inverted generates tension on the vaginal wall, bladder and the urethra. This can cause the urethra to move from its normal anatomic location, approximately 2 to 3 cm inferior to the clitoris, to a sub-symphyseal location making it difficult to locate. Also, the uterine cervix if completely inverted and flush with the vagina will be difficult to identify. Sometimes complete inversion is not the case and a constricting ring, representing the cervix, can be felt.

Preoperative evaluation with magnetic resonance imaging (MRI) has been described [9]. Sagittal views demonstrate a U-shaped endometrial cavity, while axial images show a bullseye configuration.

Many surgical techniques have been described, abdominally those of Huntington and Haultain, and vaginally those of Kustner and Spinelli [6,10]. Due to concern for the location of the ureters because of the recent dilatation of the cervix, and the inability to revert the uterus, we employed an abdominal approach. We made numerous attempts to perform the Huntington technique with little success. Ultimately we used the Haultain procedure with careful recognition of bilateral ureters, and postoperative diagnostic cystoscopy.

## Conclusion

Chronic nonpuerperal inversion of the uterus is uncommon, with little more than 100 reports in the literature. Its presence should be suspected when a larger prolapsed fibroid is encountered. Biopsy of the mass is prudent given its occurrence with uterine malignancy. In chronic inversion secondary to a fibroid, infection of the fibroid and uterus should be suspected. An attempt at vaginal restoration and removal has been reported but is difficult. Abdominal hysterectomy may be necessary, taking care to locate the distal ureters, with intraoperative cystoscopy to ensure bladder and ureteral integrity.

## Competing interests

The authors declare that they have no competing interests.

## Authors' contributions

Each of the above authors has made substantial contributions to conception and design, or acquisition of data, or analysis and interpretation of data. They all have been involved in drafting the manuscript or revising it critically for important intellectual content; and have given final approval of the version to be published.

## Consent

Written informed consent was obtained from the patient for publication of this case report and accompanying images. A copy of the written consent is available for review by the Editor-in-Chief of this journal.
